# Supplementary material for: Design, Synthesis, and Biological Evaluation of N-Acylhydrazones and Their Activity Against Leishmania amazonensis Promastigotes
Source: Microorganisms. 2025 Jul 2;13(7):1563. doi: 10.3390/microorganisms13071563 (PMC12298860; doi:10.3390/microorganisms13071563)
Supplement: Supplementary file 1 [file microorganisms-13-01563-s001.zip › RMN amostras-20241114T155656Z-001.zip!/RMN amostras/composto 4 - PMM 43-17 13Ca.pdf]

PMM 43/17 - Joao Mafra
op. Charles

farmanguinhos
Instituto de Tecnologia em Fármacos

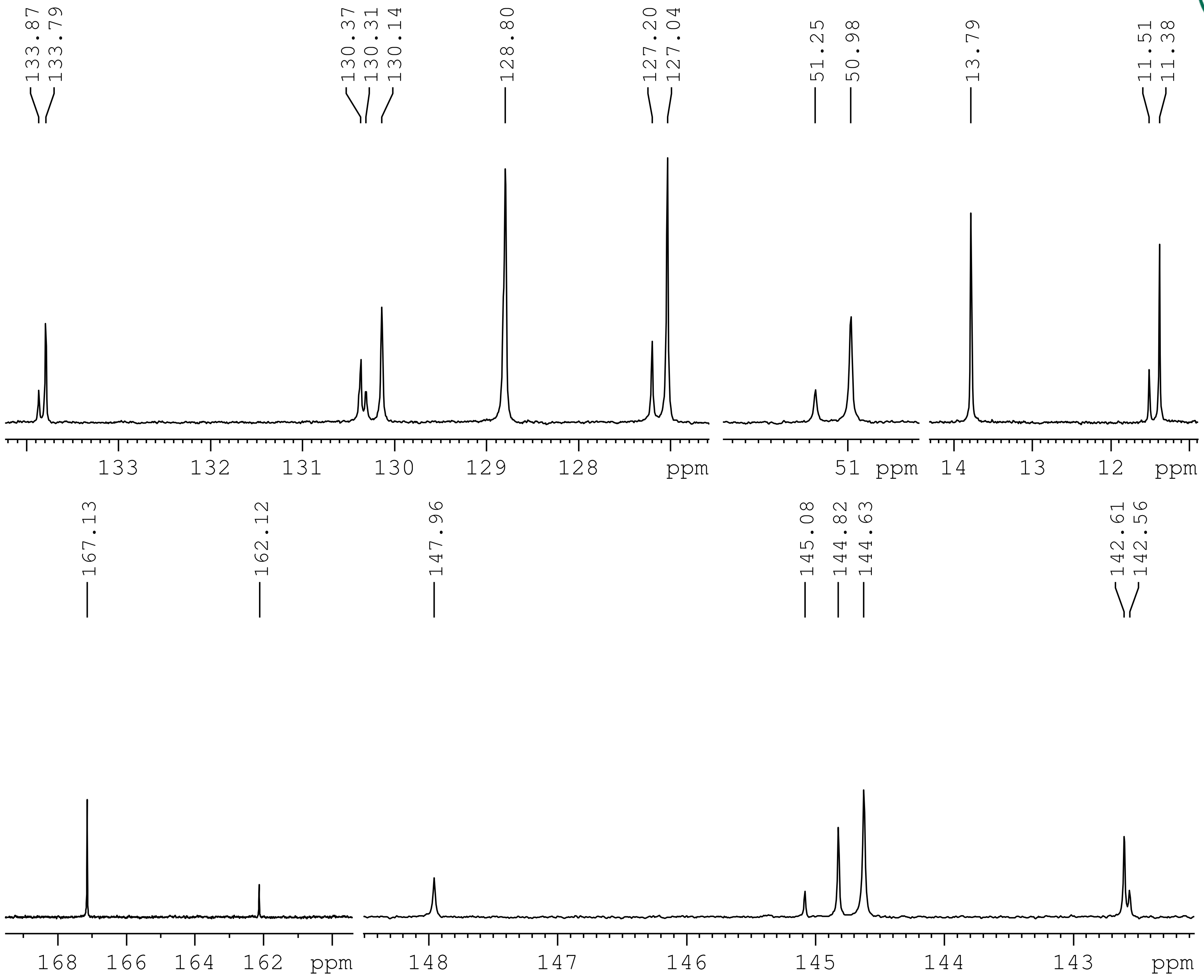

Current Data Parameters		
NAME	11-20241001T164903Z-00	
EXPNO	11	
PROCNO	1	
F2 - Acquisition Parameters		
Date_	20180124	
Time	23.48	h
INSTRUM	spect	
PROBHD	Z122623_0041 (	
PULPROG	zgpg30	
TD	65536	
SOLVENT	DMSO	
NS	4096	
DS	4	
SWH	24038.461	Hz
FIDRES	0.733596	Hz
AQ	1.3631488	sec
RG	66.74	
DW	20.800	usec
DE	18.00	usec
TE	298.0	K
D1	1.00000000	sec
D11	0.03000000	sec
TD0	1	
SFO1	100.6278593	MHz
NUC1	13C	
P1	10.00	usec
PLW1	35.50000000	W
SFO2	400.1516006	MHz
NUC2	1H	
CPDPRG[2	waltz16	
PCPD2	90.00	usec
PLW2	7.30000019	W
PLW12	0.11919000	W
PLW13	0.05995100	W
F2 - Processing parameters		
SI	32768	
SF	100.6178463	MHz
WDW	EM	
SSB	0	
LB	1.00	Hz
GB	0	
PC	1.40	
